# The Functions of the Membrana Tympani, Illustrated by Disease

**Published:** 1886-11

**Authors:** 


### *THE FUNCTIONS OF THE MEMBRANA TYMPANI ILLUSTRATED BY DISEASE.*

Sir William Dalby, in a brief but instructive article in the July issue of *The American Journal of the Medical Sciences*, points out that our knowledge of the functions of the membrana tympani may be added to by the observation of this structure when it becomes altered by disease. He points out that structural changes in the tympanic membrane, as, for instance, extensive calcareous deposit of a very extensive nature may exist without impairment of hearing. The history of cases in which there have been such deposits, with diminution of hearing shows that the patients have at some previous period suffered from inflammation within the tympanic cavity, so that the changes then wrought will sufficiently account for the failure in hearing. That the position of the obstacle to hearing is in the conducting media, and, therefore, in the tympanic cavity, and not in the nervous structure can be, in such cases, readily demonstrated by experiments with the tuning-fork.

Loss of continuity in tympanic membrane, he also shows, does not necessarily interfere with its function, provided that the ligamentous support which it affords to the chain of ossicles is not impaired.

In several instances in which the membrane has been accidentally pierced with a very sharp-pointed object, as a pin, the hearing has not been found, with the most careful tests, to be injured. In these examples the healing process occupied from three to four days.

In one case, when a sudden explosion near the ear ruptured the membrane in two places, the hearing was perfect, and the ruptures healed in a few days. On comparing the notes of other cases in which the hearing was impaired by explosions, it was found that the hearing suffered more injury when the membrane was not ruptured than when it was. It would also seem from this that the force of the explosion expended itself, partially, in rupturing the membrane, and so, in a measure, some hearing was saved. At any rate, it appears not an unfair conclusion that the loss of hearing must be due, in all cases, to damage to the nervous structures; in other words, to what, for want of a more accurate term, must be called shock.

That the loss of continuity in the tympanic membrane does not of itself interfere with its functions is still further shown by the careful and continual observation of cases in which the membrane is perforated by incision or by disease, and the author thinks that the loss of hearing is due to causes which do not include this loss of continuity in the tympanic membrane.

